# A high-performance quantum dot superluminescent diode with a two-section structure

**DOI:** 10.1186/1556-276X-6-625

**Published:** 2011-12-12

**Authors:** Xinkun Li, Peng Jin, Qi An, Zuocai Wang, Xueqin Lv, Heng Wei, Jian Wu, Ju Wu, Zhanguo Wang

**Affiliations:** 1 Key Laboratory of Semiconductor Materials Science, Institute of Semiconductors, Chinese Academy of Sciences, Beijing, 100083, China

**Keywords:** quantum dot, superluminescent diode, two-section structure, optical amplification

## Abstract

Based on InAs/GaAs quantum dots [QDs], a high-power and broadband superluminescent diode [SLD] is achieved by monolithically integrating a conventional SLD with a semiconductor optical amplifier. The two-section QD-SLD device exhibits a high output power above 500 mW with a broad emission spectrum of 86 nm. By properly controlling the current injection in the two sections of the QD-SLD device, the output power of the SLD can be tuned over a wide range from 200 to 500 mW while preserving a broad emission spectrum based on the balance between the ground state emission and the first excited state emission of QDs. The gain process of the two-section QD-SLD with different pumping levels in the two sections is investigated.

## Introduction

Superluminescent diodes [SLDs] have attracted extensive attention for a wide range of applications, such as optical coherence tomography [OCT] [1,2], optical fiber-based sensors [3-5], external cavity tunable lasers [6-8], optoelectronic systems [9], etc. A wide emission spectrum corresponding to a low degree of coherence is required for these applications of SLD, which allows the realization of sensors with improved resolution. It has been proposed that self-assembled quantum dots [QDs] [10-12] and quantum well grown on a high-index surface are beneficial to broaden the spectral bandwidth of the device [13]. Till now, QDs have successfully been used as the active media in several broadband light-emitting devices, such as QD-SLDs [14-20], QD semiconductor optical amplifiers [SOAs] [21-23], and QD broadband laser diodes [24-26]. For QD-SLD devices, a high power of 200 mW [14] and a wide spectral bandwidth of more than 140 nm [27,28] have been achieved. Most recently, an intermixed QD-SLD exhibits a power of 190 mW with a 78-nm spectral bandwidth [29].

For a typical SLD device structure with a single current-injection section, the high output power can only be obtained at a high pumping level, where the device demonstrates a narrow spectrum emitted predominantly from the QDs' excited state [ES] due to the low saturated gain of the QD ground state [GS]. It is difficult to achieve high-power and broad-emitting spectrum simultaneity. However, a high-power SLD that is broadband emitting is required in some fields. As an example, in an OCT system, a high power is usually needed to enable greater penetration depth and improve the imaging sensitivity [30]. Numerical investigation [31] and experimental evidence [32,33] have shown that this limitation can be overcome by using a multi-section structure in an SLD device, which allows the emission spectrum and output power to be tuned independently. A quantum-well SLD with a two-section structure which integrates monolithically an SLD with an SOA has been reported, which exhibits an output power that is one or two orders of magnitude higher than that in conventional SLD devices [34].

In this paper, a QD-SLD device, which has a two-section structure monolithically integrating an SLD with an SOA, is fabricated. A high power (500 mW) with a broad emission of 86 nm is obtained. By properly controlling the current injection in the two sections of the QD-SLD device, the power tunability over a wide range from 200 to 500 mW is achieved, with the preservation of a nearly constant spectral width.

## Experiment

The epitaxial structure of the QD-SLD device in this study was grown by a Riber 32P solid-source molecular beam epitaxy machine on n-GaAs(001) substrate. The epitaxial structure consists of ten InAs-QD layers separated from each other by a GaAs spacer; each of them is formed by depositing a 1.8-monolayer InAs at 480°C and covered by a 2-nm In_0.15_Ga_0.85_As. Ten QD layers plus the GaAs waveguide layers form the whole active region which is sandwiched between 1.5-μm n- and p-type Al_0.5_Ga_0.5_As cladding layers. Finally, a p^+^-doped GaAs contact layer completes the structure.

**Figure 1:**
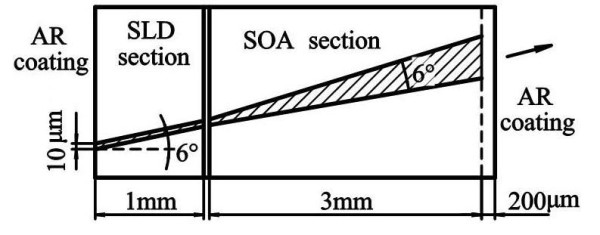
**Schematic diagram of the QD-SLD device with a two-section structure**.

A QD-SLD device with an index-guided ridge waveguide and a two-section structure was fabricated. A schematic diagram of the geometrical design (not to scale) is shown in Figure 1. The device integrates monolithically an SLD with a tapered SOA. The SLD section is 1-mm long and 10-μm wide. The tapered SOA section is 3-mm long with a full flare angle of 6°. The ridge waveguide was fabricated using photolithography and wet chemical etching. The center axis of the ridge is aligned at 6° with respect to the facet normal to suppress lasing. A 200-μm-length output window structure (no electric contact) is used to reduce the risk for catastrophic optical damage of the output facet with a high output power. Ti/Au and AuGeNi/Au ohmic contacts were evaporated on the top and back of the wafer, respectively. A 20-μm-wide separation between the SLD and the SOA sections is realized by removing the upper Ti/Au ohmic contact and the 0.5-μm epilayer using photolithography and wet chemical etching. After metallization, the device was cleaved and mounted p-side up on a copper sink using an indium solder. Antireflection coatings of λ/4 were used on both facets of the device. The QD-SLD device was characterized by light power-injection current [*P*-*I*] and electroluminescence measurements at room temperature under a pulsing (1 kHz repetition rate and 3% duty cycle) injection in the SOA section and a continuous-wave injection in the SLD section, respectively.

## Results and discussion

**Figure 2:**
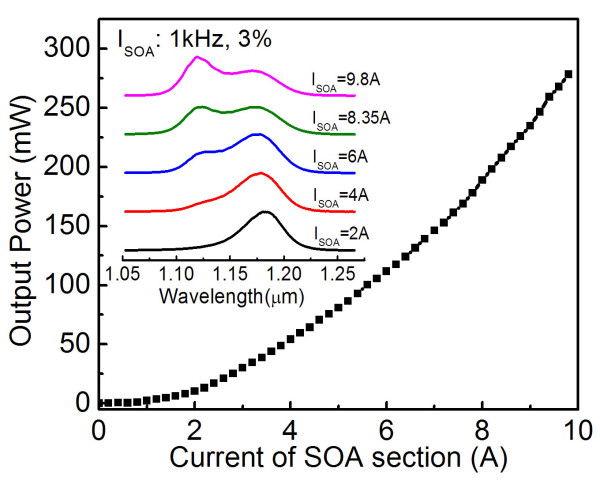
***P*-*I *characteristic of the SOA section with the SLD section un-pumped**. The inset shows the normalized emission spectra under different injection currents of the SOA section.

Figure 2 shows the *P*-*I *characteristic of the SOA section with the SLD section un-pumped and acting as a rear optical absorption region. A superluminescent characteristic is clearly observed by the superlinear increase in optical power with the current. At a current of 9.8 A, a maximum output power of 280 mW is obtained. The emission spectra under different injection currents in the SOA section [*I*_SOA_] are shown in the inset of Figure 2. When *I*_SOA _= 2 A, the center wavelength of the emission spectrum is 1.18 μm with a full width at half maximum of 43 nm, which corresponds to the QDs' GS emission. The relatively wide GS emission is attributed to the size inhomogeneity that is naturally occurring in self-assembled QDs. With the increasing *I*_SOA_, the emission spectra are clearly broadened to the short-wavelength side, which should be attributed to the sequential carrier filling into the first ES [ES1]. For a given *I*_SOA _of 8.35 A, due to the nearly identical contribution to the emission from the QDs' GS and ES1, a 94-nm broad spectrum with a power of 200 mW is achieved.

**Figure 3:**
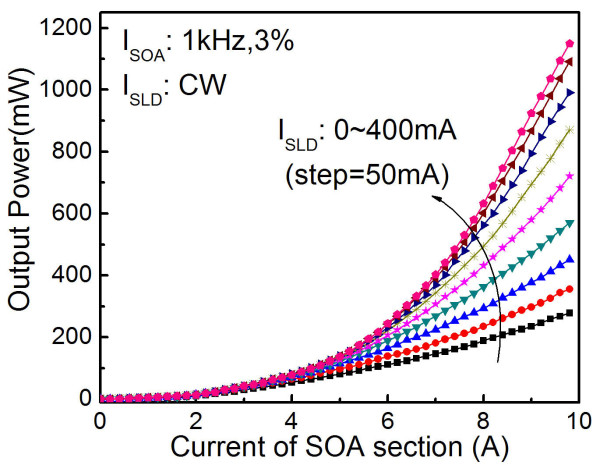
**Output power versus SOA current under different injection currents of the SLD section**.

**Figure 4:**
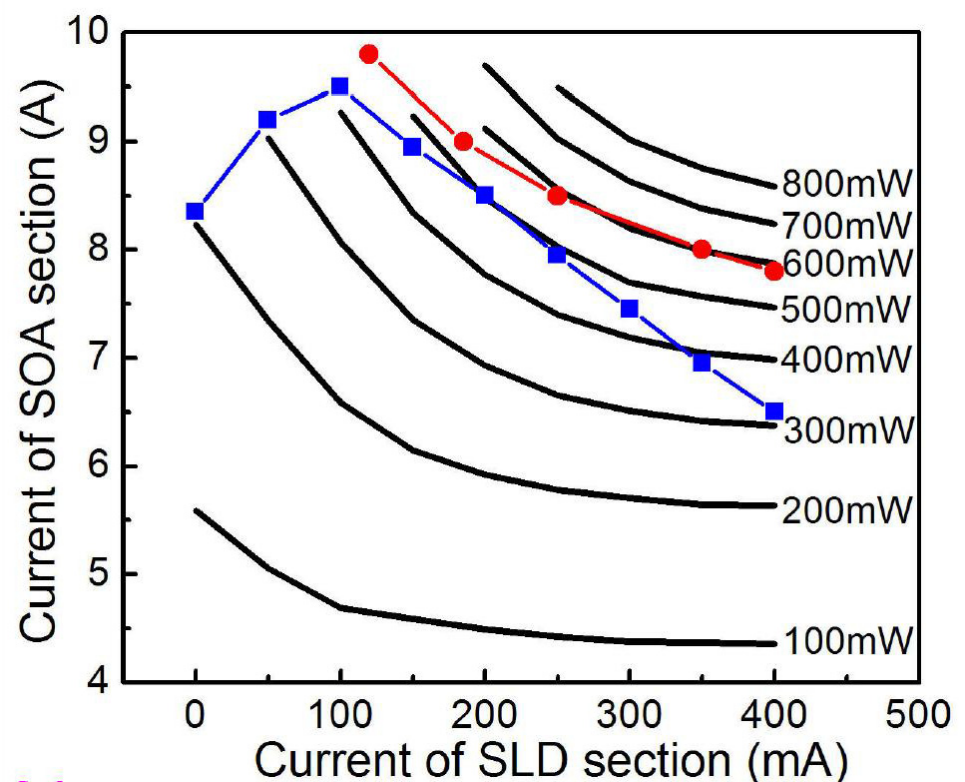
**Equal power curves (solid lines) as function of the currents injected in the two sections**. The solid squares show the combinations of currents at which QDs' GS and 1st ES give equivalent contributions to the emission spectra. Current combinations at which the device begins lasing are shown in solid circles.

The characteristics of the two-section SLD device were measured when the SLD section was pumped to seed the SOA section. The output-power characteristics versus *I*_SOA _under different SLD section currents [*I*_SLD_] are shown in Figure 3. It can be seen from the figure that the output power increases rapidly with the increasing current injection in the SLD section. Without pumping the SLD section, the output power of the device is 280 mW at *I*_SOA _= 9.8 A. The output power can reach 1.15 W at *I*_SOA _= 9.8 A and *I*_SLD _= 400 mA. The device begins lasing when the power is in the range of 500 to approximately 600 mW with various SOA and SLD current combinations (refer to Figure 4). The evident increase of output power is attributed to the amplification of the input beam while propagating forward from the narrow end to the wide end of the tapered region. With a full flare angle of 6°, the incident beam will expand freely to fill the full tapered region owing to diffraction [35]. The optical density will be reduced, which increases the saturated power.

**Figure 5:**
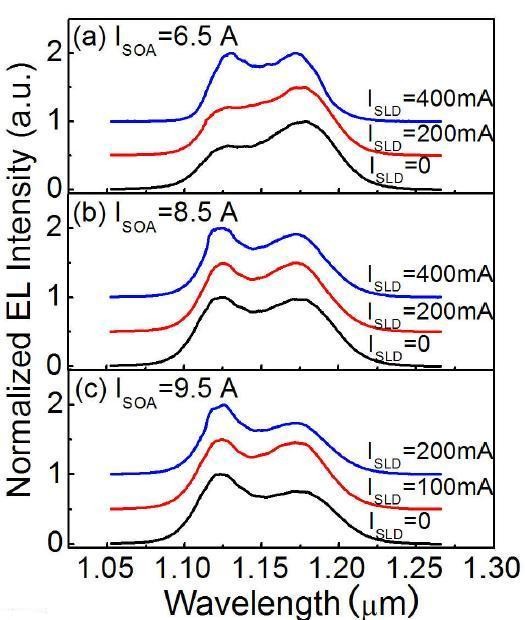
**Normalized emission spectra from the SOA facet under different pumps of the SLD section**. They are for a given SOA injection of (**a**) 6.5, (**b**) 8.5, and (**c**) 9.5 A, respectively. Some spectra are shifted vertically for clarity.

The emission spectra measured from the SOA facet under different *I*_SLD_, with *I*_SOA _fixed at 6.5, 8.5, and 9.5 A, respectively, are shown in Figure 5. As expected, it can be seen from the figure that the spectrum shape and emission bandwidth of the QD-SLD device with the two-section structure can be tuned by properly controlling the current injection in the two sections. With *I*_SOA _fixed at 6.5 A as shown in Figure 5a, the GS emission provides the main contribution to the spectrum when the SLD section is not pumped. To obtain a more broadened emission bandwidth based on the balance of the QDs' GS and ES1 emissions, the input beam from the SLD section must provide a greater amount of ES emission. When the SLD section is driven with 400 mA of current-injection in order to seed the SOA section, the resultant emission of the QDs' GS and ES1 have nearly equivalent contributions, and a 76-nm bandwidth is obtained. At this working point, the device gives a 320-mW power output. Similarly, a broad emission spectrum based on the balance between the GS emission and the ES1 emission of QDs is achieved at *I*_SLD _= 200 and 100 mA for a given *I*_SOA _of 8.5 and 9.5 A, respectively. With *I*_SOA _fixed at 8.5 A, when the SLD section is driven with a 200-mA current-injection to seed the SOA section, the QD-SLD device exhibits a broad emission spectrum of 86 nm and a simultaneous high output power of 504 mW. For a given *I*_SOA _of 9.5 A, with the SLD section un-pumped, the ES1 emission provides the main contribution to the emission spectrum. In order to achieve a balanced emission from GS and ES1, the GS-dominated emission is introduced to the SOA using *I*_SLD _= 100 mA. As a result, the resultant contribution of the QDs' GS and ES1 is equivalent. A broad emission spectrum of 88 nm with the output power of 422 mW is obtained.

It can be seen from the above results that the output power and spectrum bandwidth can be tuned by properly controlling the current densities injected in the two regions of the QD-SLD. Figure 4 shows equal power curves as function of the currents injected in the two sections. Data points (solid squares) at which the GS and ES1 have nearly identical emission intensities, corresponding to the maximum bandwidth of the emission spectrum, are also shown in Figure 4. It can be found that the output power can be tuned over a wide range of 200 to 500 mW while preserving a broad emission spectrum. The high output power and wide power tunability is due to the two-section structure which integrates a tapered SOA section. Current combinations at which the device begins lasing are also shown in Figure 4 (solid circles). Working points of the QD-SLD device can be set in the lower left region of the borderline. An optimum working point is found in the figure that the SOA current is in the 8- to approximately 8.5-A range and the SLD current is 0.2 to approximately 0.25 A, at which a 500-mW output power and an 86-nm bandwidth are achieved simultaneously.

## Conclusion

In conclusion, a high-power QD SLD with a broad bandwidth in the emission spectra is achieved by the two-section structure which monolithically integrates an SLD with a tapered SOA. Properly controlling the current densities injected in the two sections, the QD-SLD device exhibits a maximum output power above 500 mW and a simultaneously broad bandwidth of 86 nm. Also, the output power can be tuned over a wide range from 200 to 500 mW while preserving a nearly constant spectral width.

## Competing interests

The authors declare that they have no competing interests.

## Authors' contributions

XL carried out the device process, device characterization, and data analysis; participated in the experimental design; and drafted the manuscript. PJ conceived the study, participated in its design and coordination, and performed the epitaxial growth. QA participated in the data analysis. ZW participated in its design and carried out some preparative work. XL participated in the epitaxial growth. HW participated in the device process. JW participated in the device process. JW modified the draft. ZW conceived the study. All authors read and approved the final manuscript.
